# Supplementary material for: Genome-wide data suggest a revision in management of the Caspian Sea trout Salmo caspius
Source: PLoS One. 2023 Jul 20;18(7):e0287777. doi: 10.1371/journal.pone.0287777 (PMC10358921; doi:10.1371/journal.pone.0287777)

PLOS ONE

GBS-obtained data highlighted the necessity of an updated action plan for wild stocks management of Salmo caspius in the Caspian Sea

--Manuscript Draft--

**Manuscript Number:**	PONE-D-23-06704
**Article Type:**	Research Article
**Full Title:**	GBS-obtained data highlighted the necessity of an updated action plan for wild stocks management of Salmo caspius in the Caspian Sea
**Short Title:**	Genomic structure of Salmo caspius in the southern basin of the Caspian Sea
**Corresponding Author:**	Omid Jafari<br>Gorgan University of Agricultural Sciences and Natural Resources<br>Gorgan, IRAN, ISLAMIC REPUBLIC OF
**Keywords:**	Effective population size, GBS, Genetic stock identification, Inbreeding, Restocking
**Abstract:**	Salmo caspius is a cold-water fish species of the Salmonidae in the southern basin of the Caspian Sea. The occurrence of its wild populations has undergone sever reduction during the last years. In spite of the yearly restocking activity, still no progress on the recovery of its wild population has been observed. Hence, the present study was done in order to assess the efficiency of the current restocking activity in the southern Caspian basin in term of genetic diversity. DNA extracts of 32 S. caspius from four different groups were screened using 62621 GBS-obtained SNP variants. The overal genetic diversity and Fst values were 0.18 and 0.08, respectively. Considering the observed asmixture pattern and the positive values for inbreeding coeficient it seems that S. caspius suffers from its small effective population size. In order to obtain the maximum output, rehabilitation of the habitats and spawning rivers of this critically endangered species alongside with the number of breeders are of great importance.
**Order of Authors:**	Omid Jafari
	Arezo Najafikhah
	Mehrshad Zeinalabedini
	Babak Arefnezhad
	Mohsen Mardi
	Maryam Nafari
	Maryam Nasrolahpourmoghadam
**Additional Information:**	
**Question**	**Response**
**Financial Disclosure**<br><br>Enter a financial disclosure statement that describes the sources of funding for the work included in this submission. Review the submission guidelines for detailed requirements. View published research articles from *PLOS ONE* for specific examples.<br><br>This statement is required for submission and **will appear in the published article** if the submission is accepted. Please make sure it is accurate.	The name of the funder is mentioned in the end of the manuscript.

Powered by Editorial Manager® and ProduXion Manager® from Aries Systems Corporation

**Unfunded studies**<br>Enter: *The author(s) received no specific funding for this work.*<br><br>**Funded studies**<br>Enter a statement with the following details:<br>• Initials of the authors who received each award<br>• Grant numbers awarded to each author<br>• The full name of each funder<br>• URL of each funder website<br>• Did the sponsors or funders play any role in the study design, data collection and analysis, decision to publish, or preparation of the manuscript?<br>• **NO** - Include this sentence at the end of your statement: *The funders had no role in study design, data collection and analysis, decision to publish, or preparation of the manuscript.*<br>• **YES** - Specify the role(s) played.<br><br>* typeset	
**Competing Interests**<br><br>Use the instructions below to enter a competing interest statement for this submission. On behalf of all authors, disclose any competing interests that could be perceived to bias this work—acknowledging all financial support and any other relevant financial or non-financial competing interests.<br><br>This statement is required for submission and **will appear in the published article** if the submission is accepted. Please make sure it is accurate and that any funding sources listed in your Funding Information later in the submission form are also declared in your Financial Disclosure statement.<br><br>View published research articles from *PLOS ONE* for specific examples.	No

Powered by Editorial Manager® and ProduXion Manager® from Aries Systems Corporation

**NO authors have competing interests**<br><br>Enter: *The authors have declared that no competing interests exist.*<br><br>**Authors with competing interests**<br><br>Enter competing interest details beginning with this statement:<br><br>*I have read the journal's policy and the authors of this manuscript have the following competing interests: [insert competing interests here]*<br><br>* typeset	
**Ethics Statement**<br><br>Enter an ethics statement for this submission. This statement is required if the study involved:<br><br>• Human participants<br>• Human specimens or tissue<br>• Vertebrate animals or cephalopods<br>• Vertebrate embryos or tissues<br>• Field research<br><br>Write "N/A" if the submission does not require an ethics statement.<br><br>General guidance is provided below. Consult the submission guidelines for detailed instructions. **Make sure that all information entered here is included in the Methods section of the manuscript.**	The animal study was reviewed and approved by the Ethics Committee of Agricultural Biotechology Reasearch Institute of Iran.

Powered by Editorial Manager® and ProduXion Manager® from Aries Systems Corporation

**Format for specific study types**<br><br>**Human Subject Research (involving human participants and/or tissue)**<br>• Give the name of the institutional review board or ethics committee that approved the study<br>• Include the approval number and/or a statement indicating approval of this research<br>• Indicate the form of consent obtained (written/oral) or the reason that consent was not obtained (e.g. the data were analyzed anonymously)<br><br>**Animal Research (involving vertebrate animals, embryos or tissues)**<br>• Provide the name of the Institutional Animal Care and Use Committee (IACUC) or other relevant ethics board that reviewed the study protocol, and indicate whether they approved this research or granted a formal waiver of ethical approval<br>• Include an approval number if one was obtained<br>• If the study involved *non-human primates*, add *additional details* about animal welfare and steps taken to ameliorate suffering<br>• If anesthesia, euthanasia, or any kind of animal sacrifice is part of the study, include briefly which substances and/or methods were applied<br><br>**Field Research**<br><br>Include the following details if this study involves the collection of plant, animal, or other materials from a natural setting:<br>• Field permit number<br>• Name of the institution or relevant body that granted permission	
**Data Availability**<br><br>Authors are required to make all data underlying the findings described fully available, without restriction, and from the time of publication. PLOS allows rare exceptions to address legal and ethical concerns. See the PLOS Data Policy and FAQ for detailed information.	No - some restrictions will apply

Powered by Editorial Manager® and ProduXion Manager® from Aries Systems Corporation

A Data Availability Statement describing where the data can be found is required at submission. Your answers to this question constitute the Data Availability Statement and **will be published in the article**, if accepted.<br><br>**Important:** Stating 'data available on request from the author' is not sufficient. If your data are only available upon request, select 'No' for the first question and explain your exceptional situation in the text box.<br><br>Do the authors confirm that all data underlying the findings described in their manuscript are fully available without restriction?	
**Describe where the data may be found in full sentences. If you are copying our sample text, replace any instances of XXX with the appropriate details.**<br><br>• If the data are **held or will be held in a public repository**, include URLs, accession numbers or DOIs. If this information will only be available after acceptance, indicate this by ticking the box below. For example: *All XXX files are available from the XXX database (accession number(s) XXX, XXX.).*<br>• If the data are all contained **within the manuscript and/or Supporting Information files**, enter the following: *All relevant data are within the manuscript and its Supporting Information files.*<br>• If neither of these applies but you are able to provide **details of access elsewhere**, with or without limitations, please do so. For example:<br><br>*Data cannot be shared publicly because of [XXX]. Data are available from the XXX Institutional Data Access / Ethics Committee (contact via XXX) for researchers who meet the criteria for access to confidential data.*<br><br>*The data underlying the results presented in the study are available from (include the name of the third party*	Data will be given to anyone upon request since we are still working on these data for later publications.

and contact information or URL).<br>• This text is appropriate if the data are owned by a third party and authors do not have permission to share the data.<br><br>* typeset	
Additional data availability information:	

Manuscript Click here to access/download;Manuscript;Manuscript.docx

GBS-obtained data highlighted the necessity of an updated action plan for wild stocks management of *Salmo caspius* in the Caspian Sea

Arezo Najafikhah[1], Mehrshad Zeinalabedini[2*], Babak Arefnezhad[3], Mohsen Mardi[4], Maryam Nafari[5], Maryam Nasrolahpourmoghadam[6] and Omid Jafari[7*]

[1]Department of Biotechnology, Payame Noor University, Tehran 1599959515, Iran

[2]Department of Genomics, Agricultural Biotechnology Research Institute of Iran (ABRII), Karaj, Iran

[3]OMICS Research Group (Media Teb Gene Co. Ltd.), Tehran, Iran

[4]Seed and Plant Certification and Registration Institute of Iran, Agricultural Research, Education and Extension Organization (AREEO), Karaj, Iran

[5]Iranian Fisheries Organization, Tehran, Iran

[6]Department of Fisheries Sciences, Faculty of Natural Resources, University of Tehran, Karaj 3158777871, Iran.

[7]International Sturgeon Research Institute, Iranian Fisheries Science Research Institute, Agricultural Research, Education and Extension Organization, Rasht, Iran

*** Correspondence:**

Mehrshad Zeinalabedini
mzeinolabedini@abrii.ac.ir

Omid Jafari
jaafari.omid@yahoo.com

Abstract

Salmo caspius is a cold-water fish species of the Salmonidae in the southern basin of the Caspian Sea. The occurrence of its wild populations has undergone sever reduction during the last years. In spite of the yearly restocking activity, still no progress on the recovery of its wild population has been observed. Hence, the present study was done in order to assess the efficiency of the current restocking activity in the southern Caspian basin in term of genetic diversity. DNA extracts of 32 *S. caspius* from four different groups were screened using 62621 GBS-obtained SNP variants. The overal genetic diversity and Fst values were 0.18 and 0.08, respectively. Considering the observed asmixture pattern and the positive values for inbreeding coeficient it seems that *S. caspius* suffers from its small effective population size. In order to obtain the maximum output, rehabilitation of the habitats and spawning rivers of this critically endangered species alongside with the number of breeders are of great importance.

Keywords:

Effective population size, GBS, Genetic stock identification, Inbreeding, Restocking.

Introduction

Salmonidae, is one of the most commercially important fish family with a worldwide distribution. Many of the wild resources of Salmonidae have undergone stock enhancement programs as a consequent of sever reduction in population size [1]. *Salmo caspius* (migratory form from sea to rivers) alongside with *Salmo trutta* (resident form in rivers) are the two native species of Salmonidae in the Caspian Sea basin [2]. Lately conducted studies confirmed the separation of these two species through genetic and morphologic approaches [3, 4] against the previous report by Esmaeili, SAYYADZADEH (5) on Iranian freshwater fish. During the last decades, the capture rate of these species throughout the southern basin of the Caspian Sea have had a considerable reduction. There are several factors for this observation among which the overfishing, poaching, drought, rivers' system manipulation, water pollution and dam constructions draw more attention. It is presumed that *S. caspius*, the migratory form of *Salmo* in the southern Caspian basin, has been more affected by the anthropogenic effects compared to the resident form since the number of migratory mature adults from Sea to rivers has dramatically reduced, making it difficult for the *S. caspius* to recover its wild populations [6]. To compensate this reduction, the Iranian stock management department made a semi-artificial breeding program on this species by capturing the wild mature specimens from the rivers and releasing-back the following fingerlings to the rivers. Historically, the first experience of Caspian salmon artificial breeding backs to the 1967 in Kelardasht breeding center and since then annually more than 100, 000 fingerlings are being released to the rivers of the southern basin of the Caspian Sea. Although, based on the observed sea catch it seems that *S. caspius* has not yet been recovered properly and considered endangered at the national level. There are two morphotypes of *S. caspius* based on the seasonal breeding, namely fall and spring breeders between October-Nov and March-April, respectively, however molecular investigations did not prove the significant differentiation between these morphotypes [7].

The main goal of stock assessment and restocking programs are to identify distinct genetic stocks and keep the standing genetic variations between different populations in fish of the same species [8]. Hence, using molecular information in stock management and aquaculture has been inevitable part of any successful conservation program [9]. The genetic diversity and relationship parameters obtained from genetic data provide a powerful tool to watch-through and maintain the genetic health in the brood stocks and natural populations. In the study conducted on *Salmo trutta* using eight microsatellite loci, the importance of standing genetic diversity in the brood stock against the whirling disease of generated offspring was reported [10]. Different molecular techniques have been used in fish population studies since more than three decades ago, among which SSRs have been the most ideal markers compared to other traditional techniques. Nowadays, because of the impressive advances in sequencing technologies known as NGS, researchers are able to have accessibility to the genomic information of both model and nonmodel organisms. Genotyping-by sequencing (GBS) is a technique on the basis of genome complexity reduction by restriction enzymes which has been widely applied in fish populations studies and aquaculture activities [11, 12]. The ongoing restocking program of *S. caspius* has been based on random mating of breeders without knowledge on their genetic relationships, and concerning the small effective population size of this species the final output of the restocking can be affected. Hence, the present study was done using GBS-obtained SNP markers to assess the efficiency of the current restocking activity of *Salmo caspius* in the breeding center across the southern basin of the Caspian Sea.

Materials and methods

Sampling

A total of 32 fin tissues were randomly collected from wild and captivated brood stocks of *S. caspius* in Kelardasht center in 2018 (**Fig 1**). The specimens were from four groups named as NCP (New Candidate Parents); CPLY (Candidate Parents Last Year); CO (Candidate offspring) and CP

(Captured parents). The fin tissue samples were stored in absolute ethanol and transferred to the genetic lab of Systems Biology department at ABRII for the later use in molecular studies.

Fig 1. Restocking center of Caspian salmon at Kalardasht, Iran

DNA Extraction

The DNA extraction of the fin tissue samples was done with Qiagen DNeasy Blood and Tissue kit using the provided guideline by the manufacturer. After the DNA extraction, quality in terms of integrity and pollution and quantity of the extracted DNA samples were assessed by Nano drop ND-1000 and 1% agarose gel electrophoresis.

GBS library preparation and sequencing

The method for GBS library preparation and sequencing is fully described by Jafari, Zeinalabedini (11) in their paper on common carp. The paired-end GBS sequencing of the samples was done at BGI company in China. Briefly, the Apek I enzyme (NEB, USA) was used to digest 100ng of each DNA sample. The specific common and barcode adapters were ligated to the produced DNA fragments and incubated at 22 degrees of centigrade for 1h. In the next step, equal volumes of the DNA products from each sample were pooled and purified using the QIAquick PCR Purification kit (Qiagen). The enrichment of adapter-ligated fragments was done through PCR amplification using PCR Primer Cocktail and PCR Master Mix. PCR products between 180-480bp were selected on an agarose gel. Agilent Technologies 2100 Bioanalyzer and the ABI StepOnePlus Real-Time PCR System were used for quality control assessment of the libraries. In the end, the 150bp PE sequencing of the GBS libraries was done in a lane of Illumina Hiseq 2000 at BGI.

Bioinformatic analyses

Quality filtering and SNP calling

The FASTQC ver. 0.11.5 was hired for quality control of the fastq files (Babraham Bioinformatics, Babraham Institute (www.bioinformatics.babraham.ac.uk/projects/fastqc/). Stacks pipeline version 2 [13] was implemented for the following SNP calling. The process rad tag package with the default parameters was used for raw reads filtering and omitting the low-quality reads. The clean reads were then mapped to the recent genome assembly of *salmo trutta* (NCBI; GCF_901001165.1) using Bowtie2 ver. 2.3.2 with activating the --very-sensitive option [14]. The sorted BAM files were fed into gstacks to build loci from the PE reads, remove the PCR duplicates and call the SNPs. Finally, the Populations module of the Stacks 2 was used to generate a Variant Call Format (VCF) file with the loci available in at least 65% of the animals (-r 0.65), filtering possible sequencing errors, paralogous sequence variants (PSVs) and uninformative polymorphisms. HWE and ld filtering were done through the GBS_SNP_Filter package [15]. Regarding the ld treatment, merely one locus of the loci in linkage dissequilibrium (with $r^2 > 0.5$) was remained in the final vcf file. The PGDSpider 2.1.1.5 software was used to convert the generated VCF file into required formats [16].

Population structure analysis and Genetic diversity

Three different analytical methods were used for cluster analysis. Firstly, an individual-based principal component analysis (PCA) was done in R [17] package ape [18]. The dispersal of the fish individuals was visualized using the first two components by ggplot2 package [19]. Secondly, a Discriminant Analysis of Principal Components (DAPC) was done on the SNP data aiming to maximize the variance among groups. Thirdly, the structure analysis was performed in R using adegenet package [20], considering the lowest BIC for estimating the number of optimum populations (K parameter).

The Nei-based genetic distances among individuals and different groups were calculated using the poppr package [21]. The fineRADSTRUCTURE was also used to evaluate the level of genetic structuring based on shared coancestry [22, 23]. The Fst index between different groups of *S. caspius*

was extracted using Genepop 4.0.9 [24]. Observed (Ho) and expected heterozygosity (He) indices were taken using package GBS_SNP_Filter.

Results

The final VCF file contained 62621 variant SNPs after different steps of filtering. PC1 and PC2 showed 9.63 per cent of the total variance and the PCA results based on the two first components illustrated that the fish individuals of the NCP had a wider dispersal and were far away from the other groups (**Fig 2**). Similar to the PCA, the DAPC scatter plot separeted the NCP group from the other *S. caspius* individuals (**Fig 3**). The genetic background of the investigated fish individuals revealed a high level of admixture structure for the all investigated fish specimens except for the NCP (**Fig 4**).

Fig 1. PCA scatter plot of *S. caspius* brood stocks based on the two first components (CO: Candidate offspring, CP: Captured parents, CPLY: Candidate parents, NCP: New candidate parents)

Fig 2. DAPC scatter plot of investigated brood stocks of *S. caspius*

Fig 3. The admixture plot showing the proportion of genome shared between different clusters. The X-axis shows all the fish individuals in this study and the Y-axis represents the proportions of the individuals' genome belonging to different ancestral populations.

The observed heterozygosity (Ho) as one of the indices of genetic diversity had its maximum value in the NCP group (Ho= 0.30), while the CO group had the lowest value with Ho= 0.12 (**Table 1**). The maximum number of private alleles were identified in the CPLY group while the NCP showed the less regarding this index. The highest and lowest rate of inbreeding values were recognized in the CO and NCP groups with Fis values of 0.72 and 0.24, respectively. Details information of genetic divesity paramters obtained from SNP data on *S. caspius* are available in **Table 1**. The pairwise Fst values for the between group comparisons ranged from 0.04 to 0.16 for CPLY-CO and NCP-CP comparisons, respectively (**Table 2**). Nei-based genetic diversity showed the maximum genetic diversity between NCP and CP groups (**Fig 5A**), however, we observed some indications of within-population differentiation using fineRADSTRUCTURE analysis to identify fine-scale genetic clustering. Accordingly, the fineSTRUCTURE classification based on the common shared genome among individualas (coancestry history) detected three main and seven sub-groups of *S. caspious* (**Fig 5B**).

Table 1. Genomic information obtained on the brood stocks of *S. caspius*

	NCP	CPLY	CO	CP
Number of Private alleles	823	2107	1809	1278
Observed heterozygosity	0.30	0.13	0.12	0.19
Observed homozygosity	0.7	0.87	0.88	0.81
Expected heterozygosity	0.34	0.36	0.37	0.33
Expected homozygosity	0.66	0.64	0.63	0.67
Nucleotide diversity	0.43	0.39	0.39	0.38
Inbreeding coefficient	0.24	0.65	0.72	0.41

Table 2. Fst index comparisons between different brood stocks of *S. caspius*

	NCP	CPLY	CO	CP
NCP	-	0.096185	0.063366	0.163711
CPLY		-	0.044616	0.086125
CO			-	0.060477
CP				-

Fig 4. A) The UPGMA phylogenetic tree using the nei-based genetic distances between different brood stocks of *s. caspius*. **B)** fineSTRUCTURE classification based on the obtained SNP markers on *S. caspius* in the Tunkabon breeding Center. Individuals classified along the heatmap's diagonal have common shared coancestry histories and pairwise comparisons outside the diagonal illustrates level of shared coancestry between groups of *S. caspius*.

Discussion

In the present study, the genomic structure of *Salmo caspius* using 62621 SNP markers was investigated at the Kelardasht breeding center in Iran. To the best of our knowledge, this is the first study in the southern basin of the Caspian Sea which aimed to unraveal the genomic structure of *S. caspius* in the restocking center with a high number of genetic markers. The obtained information based on the SNP markers suggets an especial genetic management of the brood stocks of *S. caspius* in the Iranian Restocking center of *S. caspius*.

Population structure and genetic diversity indices

A high level of admixture pattern was observed in the studied candidates of *S. caspius*. Based on the structural analyses, all investigated fish individuals of *S. caspius* in this study were classifid in two genetically distict populations, so that the NCP group (New candidate parents) could be considered in one clade and the other fish candidates can be placed into the second branch. The Fst as an index of populations' differentiation ranged from 0.04 to 0.16. According to Wright (25), the Fst value

between 0.0- 0.05 is an indication for the low differentiation, the Fst between 0.05- 0.15 indicates a mediocre divergence while the Fst above 0.15 implies a complete separation of the populations. Based on the obtained values of Fst index in this study, populations are in medium level of differentiation with the mean Fst value of 0.08. Having said that, the fish candidates from the NCP group are completely separated from the other candidates with Fst higher than 0.15, showing that the wild source of *S. caspius* can impose a satisfactory level of genetic diversity but the number of brood stocks in each breeding activity should increase to boast the effective population size and consequent genetic diversity in the next generation.

Considering the genetic diversity, all investigated populations showed a low level of genetic diversity based on the observed heterygosity wherase the NCP group showed the highest heterozygosity. Previously conducted studies on the wild Caspian trout identified different populations of *S. caspius* in two different rivers however they reported a realtive low nucleotide diversity based on D-loop region of mtDNA [26]. In the study on seven different wild and hatchery-based populations of *S. caspius* a satisfactory level of genetic diversity was reported based on the mtDNA control region and nuclear markers (ITS-1 and 10 microsatelite loci), however, it was clearly reported that the current restocking program has not produced detectable genetic changes in the wild populations [27]. The results obtained through the present study that the ongoing process of Caspian trout restocking has produced low to medium level of genetic differentiation, suggesting an especial attention for expanding the effective population size during the artificial breeding programs to replenish the genetic diversity of *S. caspius* in the southern basin of the Caspian sea.

The current restocking programe of *S. caspius* is mainly baised on the maiting of the wild captured parrents in different years (usually two years in a row), then producing brood stock candidates from this mating and in the end using theses produced F0 candidates for breeding schemes with the wild counterparts. Based on the obtained results in this study it seems that the genetic diversity during the undergoing restocking activity of *S. caspius* decreses only after one generation. As it is observed in

the CO group, the level of genetic diversity of the candidates offsprings is three times smaller than the new candidate parents from the wild source. On the other hand, positive values for the inbreeding coeficient was observed in the all studied groups, indicating small effective population size both in wild and hatchery-generated populations which results inbreeding depression. Caspian brown tout has been placed in the CR level (Critically endangered) according to the IUCN red-list [6, 28, 29], and in accordance with the obtained results in this study it seems that there is a great need to review the restocking activities in order to heal up the wild populations of this species. Restocking is not solely a program based on artificial breeding and other considerations such as habitat restoration, recovering natural nursury grounds and providing the connectivity among populations across Caspian river basins to enhance the wild populations should be taken into consideration [27]. This integrated conservation strategy is needed as long as the combination of stocking and destruction of trout habitats probably have led to a loss of genetically distinct populations and still is ongoing. Loss of genetyic diversity can diminish the adaptive potential of *S. caspius* which severly threaten its future existence in the Caspian Sea and its related rivers [30].

Conclusion

The genomic population structure of *Salmo caspius* brood stock in the southern Caspian Sea breeding ceneter was investigated for the first time using 62621 GBS-obtained SNP markers. The obtained results through this study highlighted that current breeding strategy has maintained the low to mdeium level of genetic differentiation for *S. caspius* in the southern basin of the Caspian Sea. Positive values were observed for the inbreeding coeficients in all brood stocks is an implication for the small effective population size and a consequence of artificial breeding using realtives.

Data Availability Statement

The datasets presented in this study will be available on request.

Ethics Statement

The animal study was reviewed and approved by the Ethics Committee of Agricultural Biotechology Reasearch Institute of Iran.

Authors contribution

AN, MZ, and OJ designed the study. AN carried out the related field work, lab work, and also wrote the manuscript. BA, MZ, OJ, MNP and MN participated in data analysis. OJ and MNP interpreted the obtained results. OJ, MZ and MM reviewed and edited the manuscript. All authors contributed to the article and approved the submitted version.

Funding

We are grateful to Agricultural Research, Education and Extension Organization (AREEO) for providing the required financial and technical resources.

Conflict of Interest

BA was employed by OMICS Research Group (Media Teb Gene Co. Ltd.). The remaining authors declare that the research was conducted in the absence of any commercial or financial relationships that could be construed as a potential conflict of interest.

References

1. Hong D, Joo G-J, Jung E, Gim J-S, Seong KB, Kim D-H, et al. The Spatial Distribution and Morphological Characteristics of Chum Salmon (Oncorhynchus keta) in South Korea. Fishes. 2022;7(1):27.
2. Esmaeili H, Coad B, Gholamifard A, Nazari N, Teimory A. Annotated checklist of the freshwater fishes of Iran. Zoosystematica Rossica. 2010;19(2):361-86.
3. Salehi M, Mousavi-Sabet H, Eagderi S. The Iranian native salmonids (Salmo caspius and S. trutta), distinct species or two forms of the same species; a geometric-morphometric based hypothesis. Aquaculture Sciences. 2022;10(2):25-34.
4. Jouladeh-Roudbar A, Ghanavi HR, Doadrio I. Ichthyofauna from Iranian freshwater: Annotated checklist, diagnosis, taxonomy, distribution and conservation assessment. Zoological Studies. 2020;59.
5. Esmaeili HR, SAYYADZADEH G, EAGDERI S, ABBASI K. Checklist of freshwater fishes of Iran. FishTaxa. 2018;3(3):1-95.
6. Niksirat H, Abdoli A. On the status of the critically endangered Caspian brown trout, Salmo trutta caspius, during recent decades in the southern Caspian Sea basin (Osteichthyes: Salmonidae). Zoology in the Middle East. 2009;46(1):55-60.
7. Habibi E, Kalbassi MR, Hosseini SJ, Qasemi SA. Feasibility of identification of fall and spring migrating Caspian trout (Salmo trutta caspius) by using AFLP molecular marker. Turkish Journal of Fisheries and Aquatic Sciences. 2013;13(2).
8. Larson WA, Seeb LW, Everett MV, Waples RK, Templin WD, Seeb JE. Genotyping by sequencing resolves shallow population structure to inform conservation of Chinook salmon (Oncorhynchus tshawytscha). Evolutionary Applications. 2014;7(3):355-69.
9. Wirgin II, Waldman JR. What DNA can do for you. Fisheries. 1994;19(7):16-27.
10. Eszterbauer E, Forró B, Tolnai Z, Guti CF, Zsigmond G, Hoitsy G, et al. Parental genetic diversity of brown trout (Salmo trutta m. fario) brood stock affects offspring susceptibility to whirling disease. Parasites & vectors. 2015;8(1):1-9.
11. Jafari O, Zeinalabedini M, Robledo D, Fernandes JMdO, Hedayati A-A, Arefnezhad B. Genotyping-by-Sequencing reveals the impact of restocking on wild common carp populations of the Southern Caspian Basin. 2022.
12. Elshire RJ, Glaubitz JC, Sun Q, Poland JA, Kawamoto K, Buckler ES, et al. A robust, simple genotyping-by-sequencing (GBS) approach for high diversity species. PloS one. 2011;6(5):e19379.
13. Catchen J, Hohenlohe PA, Bassham S, Amores A, Cresko WA. Stacks: an analysis tool set for population genomics. Molecular ecology. 2013;22(11):3124-40.
14. Langmead B, Salzberg SL. Fast gapped-read alignment with Bowtie 2. Nature methods. 2012;9(4):357-9.
15. Alexander A. GBS_SNP_filter v1.x.x. . Available online at: https://githubcom/laninsky/GBS_SNP_filter (accessed January, 2021). 2018.
16. Lischer HE, Excoffier L. PGDSpider: an automated data conversion tool for connecting population genetics and genomics programs. Bioinformatics. 2012;28(2):298-9.
17. Team RC. R: A language and environment for statistical computing. 2013.
18. Paradis E, Schliep K. ape 5.0: an environment for modern phylogenetics and evolutionary analyses in R. Bioinformatics. 2019;35(3):526-8.
19. Wickham H. Elegant graphics for data analysis (ggplot2). Applied Spatial Data Analysis R. 2009.
20. Jombart T. Analyses multivariées de marqueurs génétiques: développements méthodologiques, applications et extensions: Lyon 1; 2008.
21. Kamvar ZN, Tabima JF, Grünwald NJ. Poppr: an R package for genetic analysis of populations with clonal, partially clonal, and/or sexual reproduction. PeerJ. 2014;2:e281.
22. Lawson DJ, Hellenthal G, Myers S, Falush D. Inference of population structure using dense haplotype data. PLoS genetics. 2012;8(1):e1002453.
23. Malinsky M, Trucchi E, Lawson DJ, Falush D. RADpainter and fineRADstructure: Population Inference from RADseq Data. Molecular Biology and Evolution. 2018;35(5):1284-90. doi: 10.1093/molbev/msy023.

24. Rousset F. genepop'007: a complete re-implementation of the genepop software for Windows and Linux. Molecular ecology resources. 2008;8(1):103-6.
25. Wright S. Evolution and the genetics of populations, volume 4: variability within and among natural populations: University of Chicago press; 1984.
26. Najjar Lashgari S, Rezvni Gilkolaei S, Kamali A, Soltani M. Study of genetic diversity of wild Caspian trout Salmo trutta caspius in the Sardabrud and Astara Rivers, using D-Loop region sequencing. 2017.
27. Vera M, Sourinejad I, Bouza C, Vilas R, Pino-Querido A, Kalbassi MR, et al. Phylogeography, genetic structure, and conservation of the endangered Caspian brown trout, Salmo trutta caspius (Kessler, 1877), from Iran. Hydrobiologia. 2011;664(1):51-67.
28. Kiabi BH, Abdoli A, Naderi M. Status of the fish fauna in the South Caspian Basin of Iran. Zoology in the Middle East. 1999;18(1):57-65.
29. Abdoli A. The inland water fishes of Iran. Iranian Museum of Nature and Wildlife, Tehran. 378 p. Farsi; 2000.
30. Largiadèr C, Scholl A, Guyomard R. The role of natural and artificial propagation on the genetic diversity of brown trout (Salmo trutta L.) of the upper Rhône drainage. Conservation of endangered freshwater fish in Europe: Springer; 1996. p. 181-97.

Figure 1

Click here to access/download;Figure;Fig 1.jpg

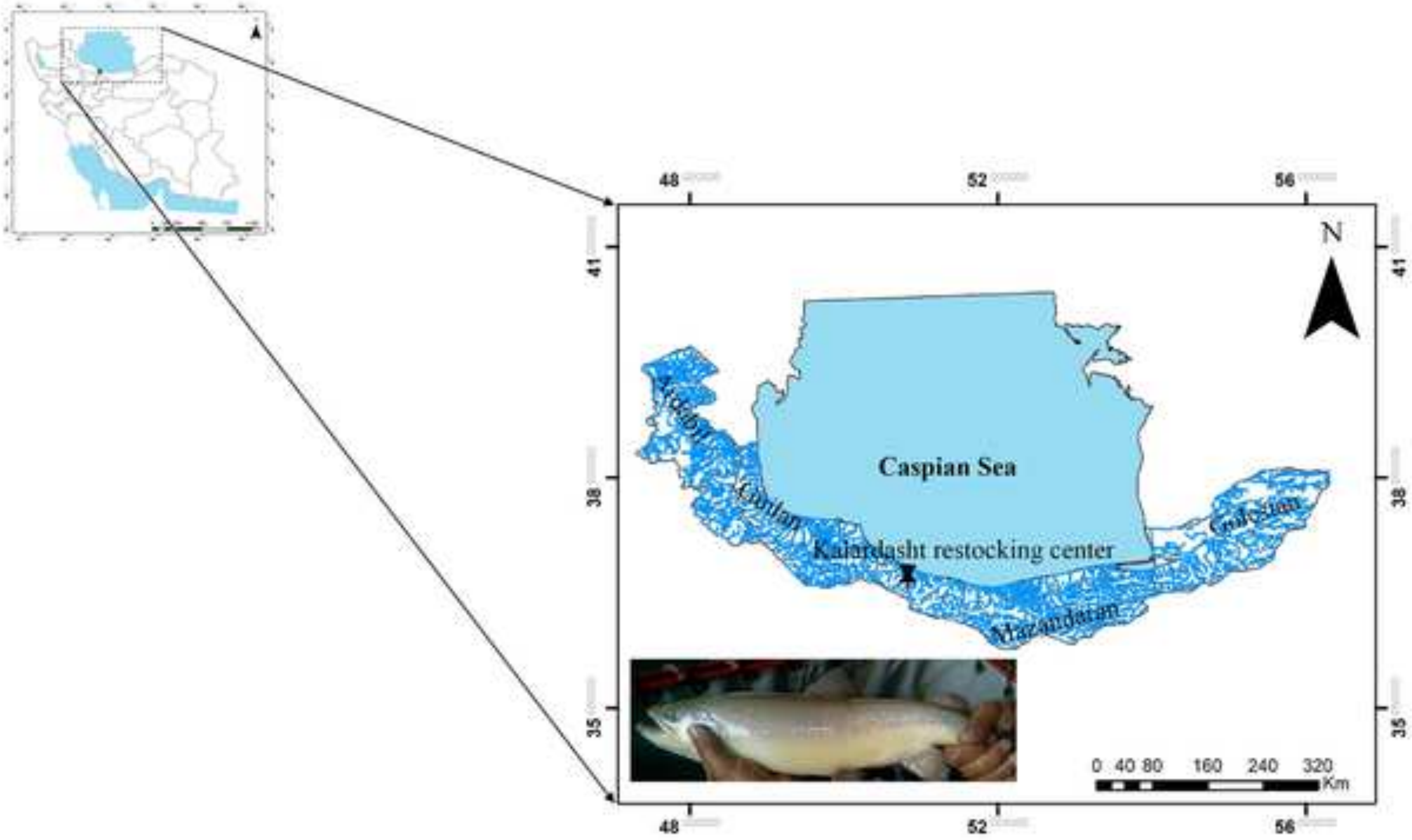

Figure 2

Click here to access/download;Figure;Fig 2.jpg

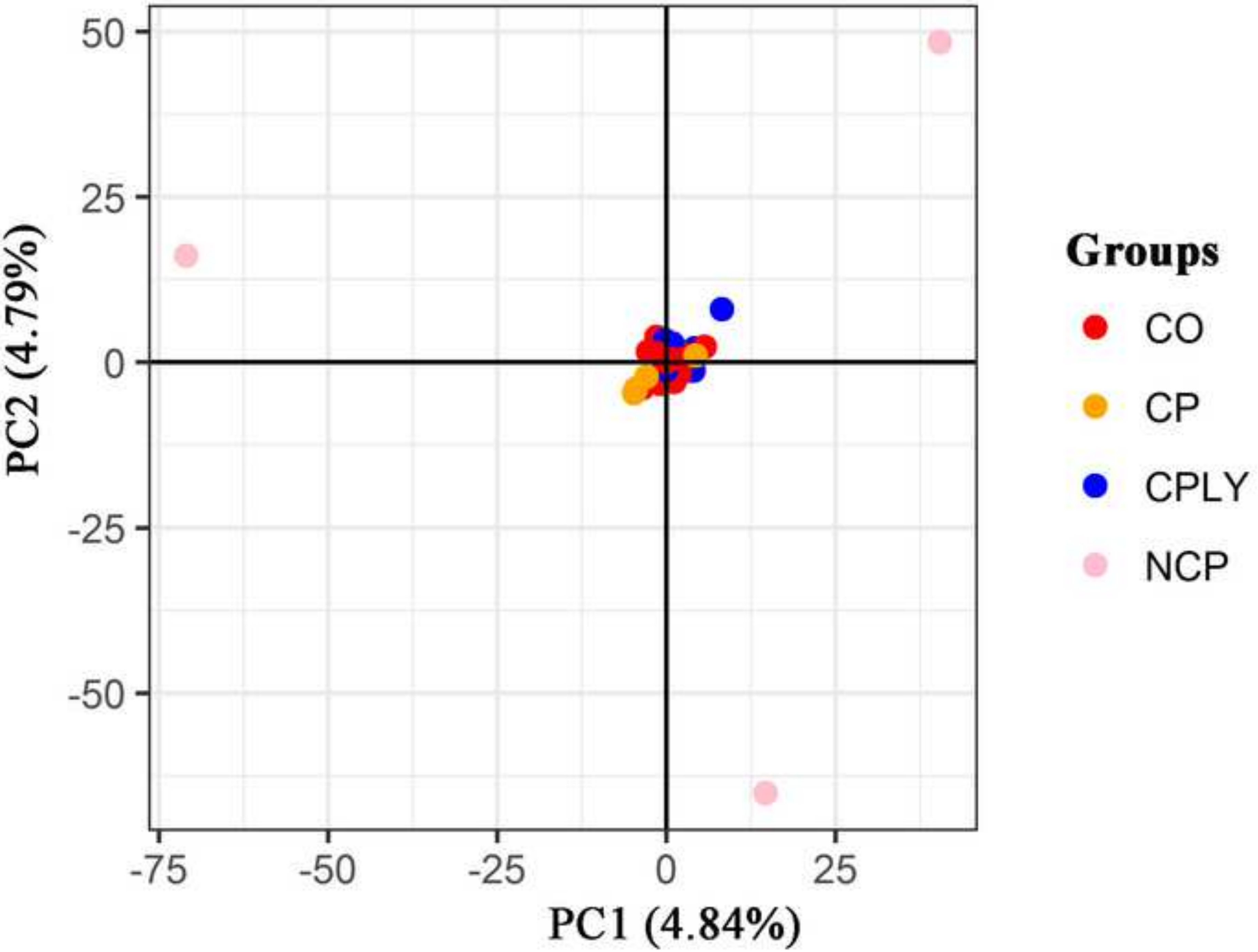

Figure 3

Click here to access/download;Figure;Fig 3.png

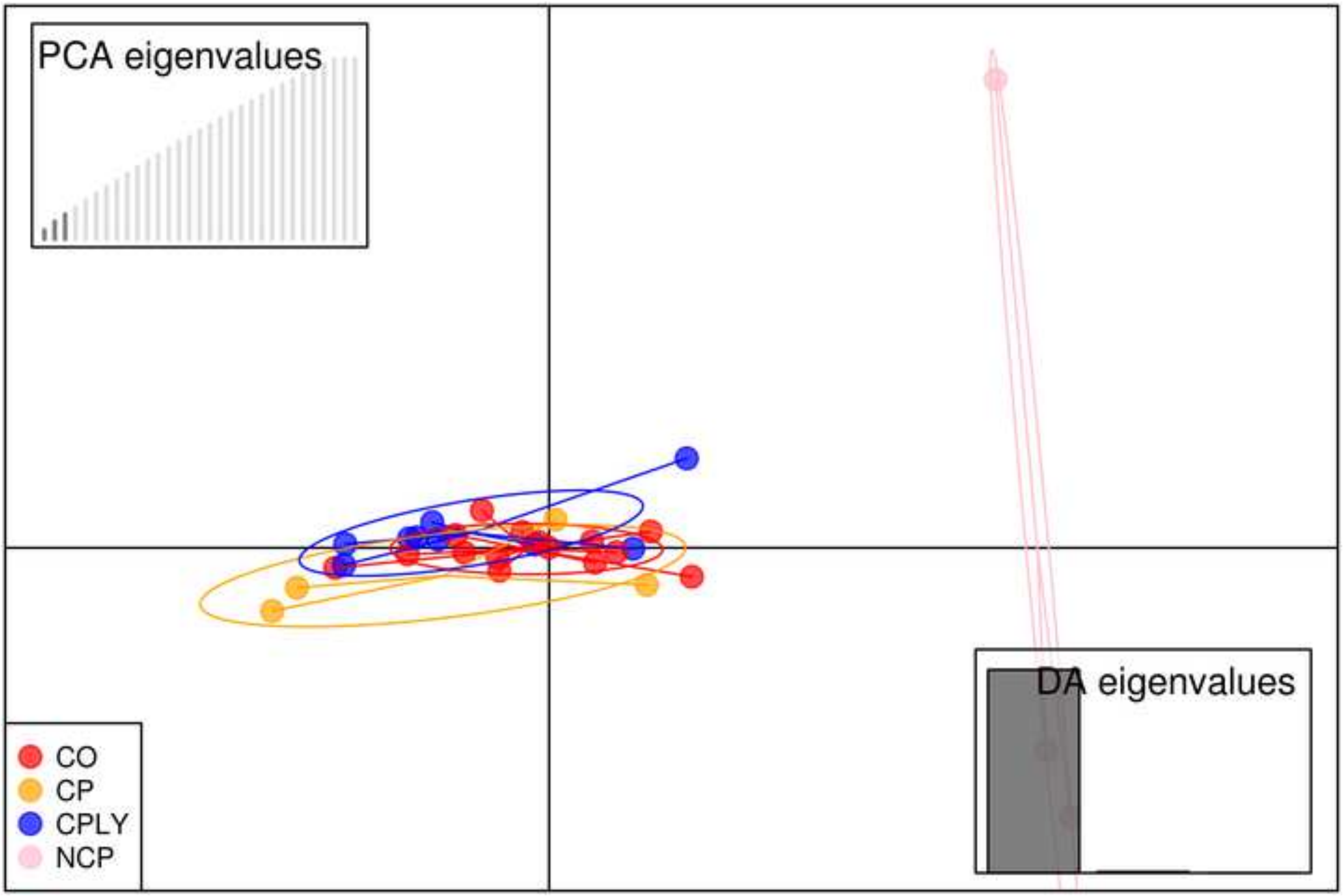

Figure 4

Click here to access/download;Figure;Fig 4.png

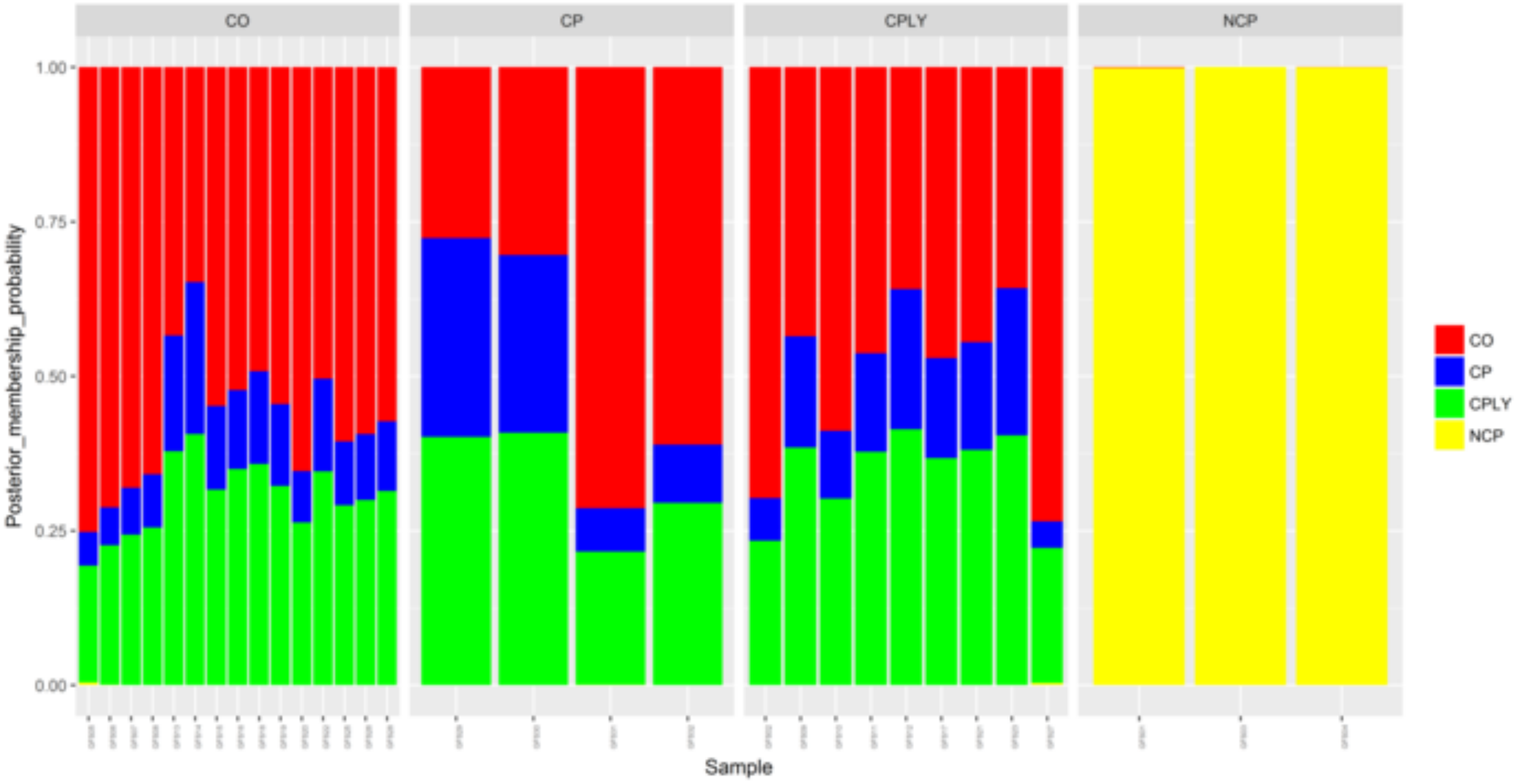

Figure 5-A

Click here to access/download;Figure;Fig 5-A.jpg

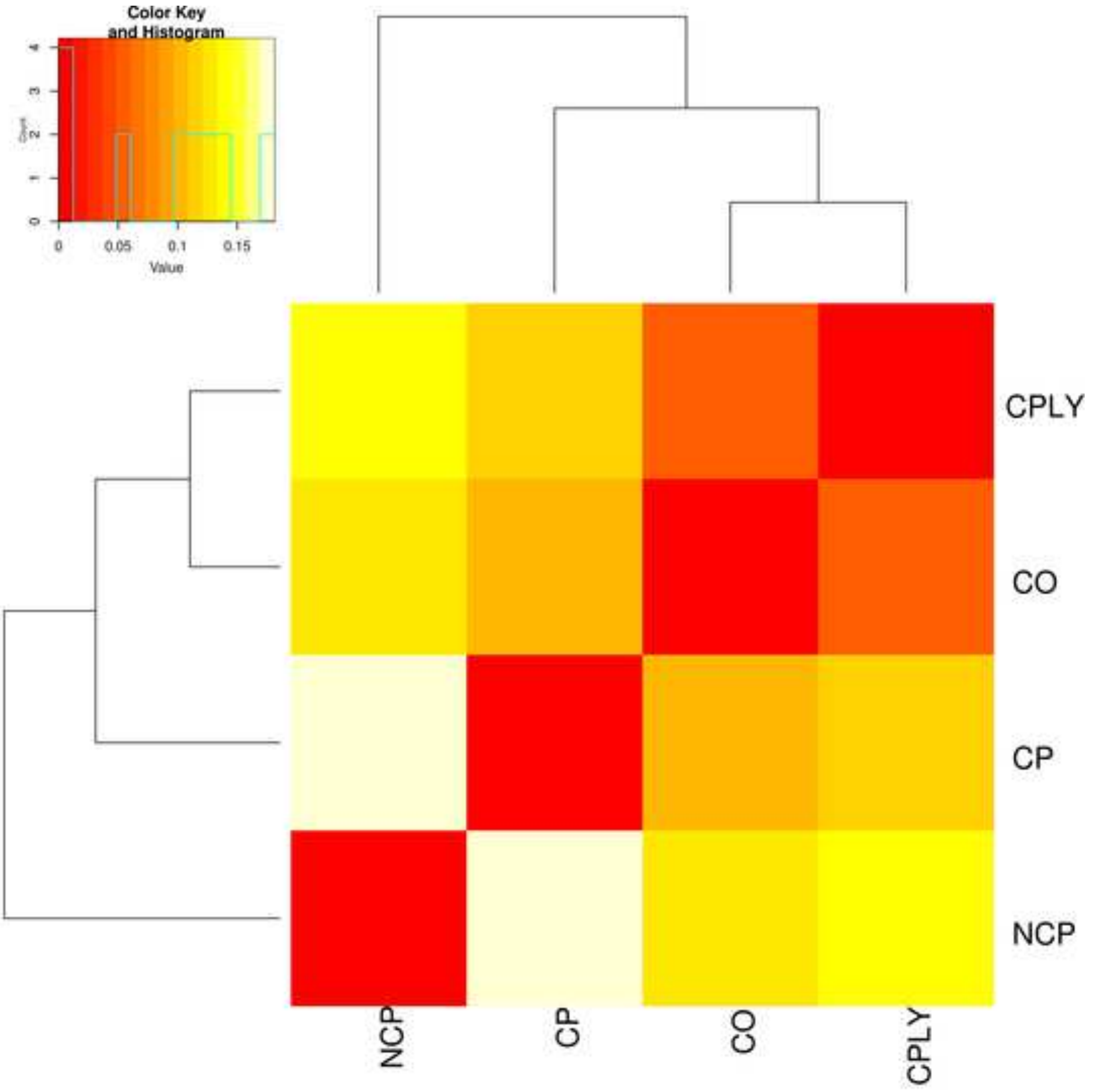

Figure 5-B

Click here to access/download;Figure;Fig 5-B.png

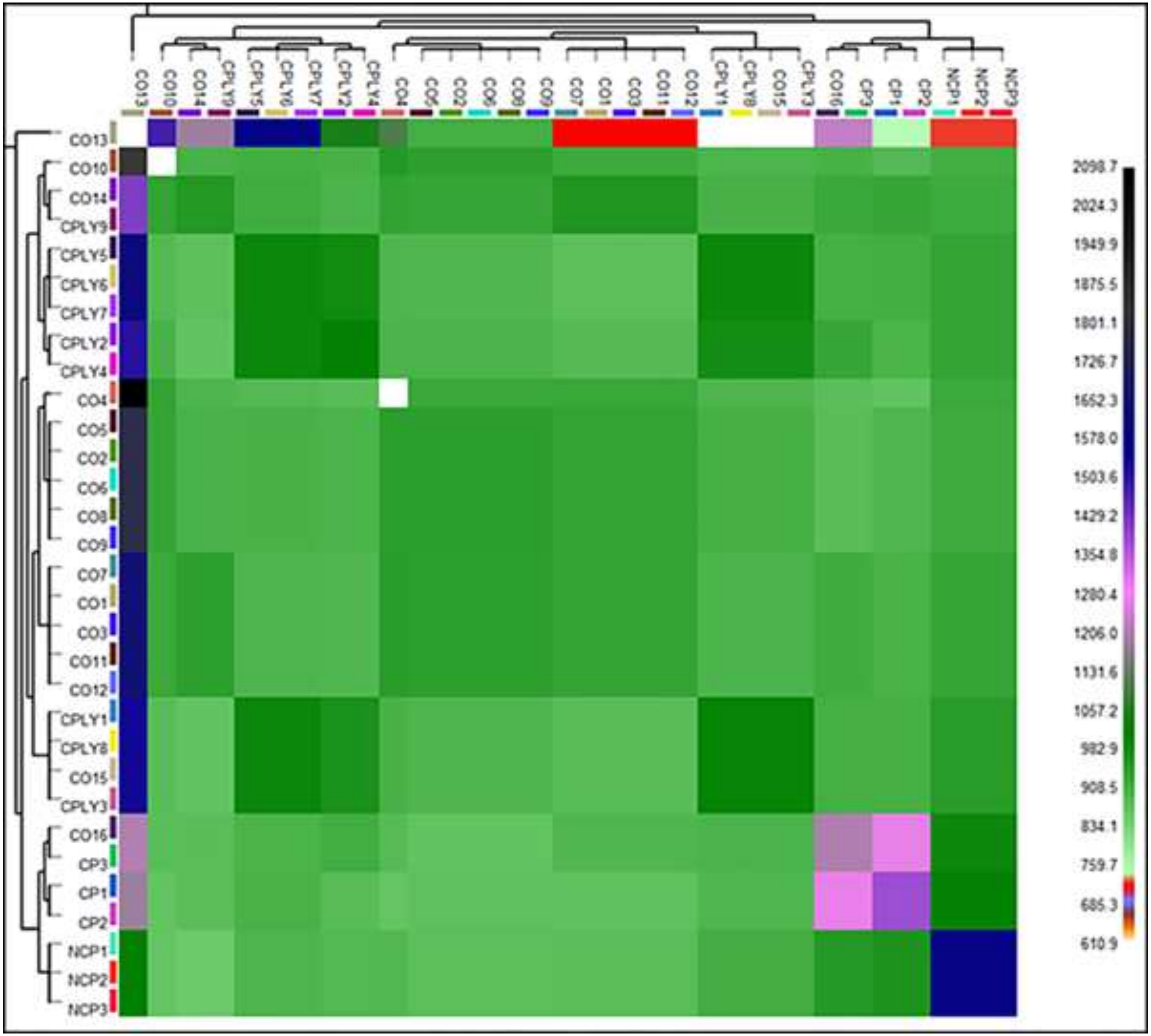

Supplement: Attachment — Submitted filename: PONE-D-23-06704_Reviewer_Comments.pdf [file pone.0287777.s002.pdf]
