# Supplementary material for: Genome-wide data suggest a revision in management of the Caspian Sea trout Salmo caspius
Source: PLoS One. 2023 Jul 20;18(7):e0287777. doi: 10.1371/journal.pone.0287777 (PMC10358921; doi:10.1371/journal.pone.0287777)
Supplement: Attachment — Submitted filename: Response to Reviewers.docx [file pone.0287777.s003.docx]

Before everything, authors are completely grateful for the peer-review of the manuscript. All comments have been seriously taken into consideration and are addressed in the manuscript.

**Reviewer 1:**

1- There are too many knowledge gaps in the introduction section (see the comments), which need further clarifications.

Response: We have made several changes and added new information into the introduction section to avoid the problem of knowledge gap.

2- The sampling section needs improvement. As a reader, I have no idea what the four study groups are (NCP, NCPLY, CO, and CP). The authors need to explain these groups in more detail, and also explain the rationale for this grouping. This part needs to be explained carefully and comprehensively, since the whole study seems to be based on it.

Response: The sampling section has been improved, specifically with the all details on the way of grouping.

3- Finally, there is a bias in the sampling. The CO and NCP groups are represented by 4 and 3 individuals respectively, whereas this is contrasted by the fact that in a standard population genetic analysis each population/group must be represented by at least 10 individuals. If possible, the number of analyzed samples should be increased for these two groups.

Response: Thank you for all your helpful comments. Unfortunately, it was not possible for us during the sampling to have more specimens because of several issues. Of course, availability of more samples can enrich the work but for example, in the year of sampling only 3 mature adults had been caught which disabled us to have more.

**Reviewer 2:**

1. The title should be revised, may be: Genome-wide data suggest a revision in management of the Caspian Sea trout Salmo caspius.

Response: The title has been changed to “Genome-wide data suggest a revision in management of the Caspian Sea trout *Salmo caspius*”

Abstract
Line 29: provide also authorship at first mention of the scientific names!

The authorship was provided: *Salmo caspius* Kessler, 1877

Line 34: GBS-obtained SNP variants: replace with genome-wide single nucleotide polymorphisms (SNP)

Response: Done.

Line 36: admixture

Response: Done.

Line 38: critically endangered? It is not even listed in IUCN redlist. Please check it!

Response: It has been modified to the nationally endangered species.

Introduction
Line 43: families

Response: Done.

Line 45: consequence

Response: Done.

Lines 45-47: no! Salmo trutta is the Atlantic trout! All natural populations of brown trout in the southern Caspian Sea basin are Salmo caspius: resident and sea-run forms are only ecological forms, but not species! Please rather than using general checklists, have a look at recent papers published on population genomics of Salmo caspius using the same approach used here.

Response: Thank you so much for the great comment. We have been referring to the published check list but now we changed to whole introduction based on the recent published papers on *salmo caspius* using GBS data.

Lines 47-49: I could not see any genetic work in references, which are cited (checklists). Please use references dealing directly with the Caspian Sea trout using robust data. It is clear that resident form of the Caspian trout or any other member of Salmo trutta species-complex is morphologically different from lake- or sea-run forms. Please see the above-mentioned references.

Response: The introduction section has been modified as you nicely suggested based on the provided references and all of them are cited now in the manuscript. Their reference numbers are: 5, 8, 9 and 11.

Lines 62-65: I guess these are not morphotypes, are they? They should be temporal ecotypes, which migrate during different seasons. Please clarify!

Response: It seems to us that the migratory behavior can be considered as a trait/morphotype, and that is why we mentioned two morphotypes. Now, it is changed to ecological forms in line 54.

Line 76: please provide the complete terms at the first mention and then use only abbreviations: Next Generation Sequencing (NGS)

Response: Done.

Lines 76-77: please provide references.
Response: Two references (17 and 18) have been mentioned in line 87.

Line 79: only two citations show the wide use of the approach in population genetics and aquaculture? Please provide examples!
Response: Some examples concerning the usage of GBS data in Fisheries sciences have been provided in lines 89-92.

Line 84: breeding center or centers across the southern Caspian Sea basin? If more than one breeding center is considered, please indicate. In m&m section authors indicate only one breeding center!

Response: Only one breeding center was considered. Indeed, Kelardasht is the one and only center for restocking *Salmo caspius* in the southern Caspian Sea.

Material and Methods

Lines 87-91: it is not clear how these fish were selected? where the fish had been caught from? Were they all from the same population? What was the relationship of different groups? What does it mean “new candidate parents”, “candidate parents last year”, “candidate offspring”, and “captured parents”? please clarify! How many specimens from each group?

Response: The new explanation for material and methods section has been provided in lines 100-106.

Line 97: not guideline, but protocol or manual

Response: Done (in line 114).

Line 98: please provide webpage of the manufacturer.

Response: Done (in lines 114 and 116).

Response:
Line 102: please refer to citations properly! Jafari et al.

Response: Done.

Line 103: webpage for BGI co.

Response: The related web page is available in line 121.

Line 114: hired? Used?

Response: “hired” was changed to “used” in line 132.

Lines 135-138: as sample sizes used in this study are small, it is suggested to use other metrics like allele frequency differences to avoid problems related to small sample size. Fst is sensitive to small sample sizes.

Response: We appreciate your very informative comments. The AFD (Allele Frequency Differences) between pairs of groups was estimated and provided now in the manuscript both in m&m section and the results (157-158, and 188-189, and **Table 2**).

Line 139: taken or calculated?

Response: "taken" was changed to "calculated".

Results:

There are no details on proportion of missing data and how it was managed! Please clarify!

Response: The proportion of missing data has been provided in supplementary information as named S12_Table. Also, during the Bioinformatic analysis, the way in which missing values were behaved has been explained in lines 160-161.

Lines 142-148: which K was selected in Structure analysis? Please clarify and provide statistical evidence.

Response: K= 2 was considered for the structure analysis based on the obtained lowest BIC. The related explanations are available in lines 151-152 and 177.

The work aims at conservation, but one of the most important statistical analyses which is mostly used in conservation genetics works is missing: I expected to see AMOVA analysis of the data to see statistical support for population structure.

Response: The AMOVA analysis has been added to the manuscript which was done using Genodive. The related sentences are in lines 158-161 and 189-190.

Line 179: Fig. 4a? appears to be 5a

Response: Done.

Discussion
Lines 186-189: please see my previous comments: it is not the first genomic work on the Caspian Sea trout. There had been other studies!

Response: Here our main focus was on the current restocking activity which is held in the Kelardasht restocking activity. So, we changed it to " To the best of our knowledge, this is the first study in the southern basin of the Caspian Sea which aimed to reveal the efficiency of the ongoing restocking activity of *S. caspius* with a high number of genetic markers."

Line 194: structure analysis

Response: Done.

Lines 196-199: Fst is prone to small sample sizes, see previous comments!

Response: As you nicely suggested, the AFD analysis has been added to the manuscript.

Line 218: based

Response: Done.

220-224: What is the mating scheme in the noted hatchery? The reduced diversity may be a result of breeding scheme! What was the sex ratio? How many brood fish had been used? And so on. All these are important factors which must be clarified.

Response: The related explanations are available in lines 254- 260.

Lines 226-227: It is not listed in the redlist as I searched! Please check!

Response: In lines 258-260 the revised sentences are provided (In spite of the fact that S. caspius has faced a drastic reduction in its wild source, still no any information is available on this species in the IUCN red list. Nevertheless, the Caspian brown trout has been considered as an endangered species in the national level).

Data availability
Line 248: data should be deposited in public databases like SRA or VCF files can be uploaded to Dryad …

Response: The raw data are deposited in SRA and now is indicated in the manuscript (PRJNA966795).

Ethics:
Lines 251-252: Was there a licence issued by the Ethics committee? Pleas provide
References

Response: The animal study was reviewed and approved by the Ethics Committee of Agricultural Biotechology Reasearch Institute of Iran (NO. 014-05-05-006-94006).

References do not follow a standard and unified format. In some cases, they are incomplete and details are missing!

Response: Thank you for your careful attention. We have used the endnote style of the Journal to avoid unwanted typo mistakes.
Figure 1 why members of the same cluster (NCP) are distributed all around the graph? Is there any justification?

Response: Concerning the high dispersal of NCP group in PCA scatter plot, it can be linked to the higher genetic diversity by NCP group, however, all other structural analysis such as fineRADstructure based on their recent shared genome put them in the same clade.
